# Decompression of a Large Periapical Lesion: A Case Report of 4-Year Follow-Up

**DOI:** 10.1155/2016/3830987

**Published:** 2016-12-12

**Authors:** Claudio Maniglia-Ferreira, Fabio de Almeida Gomes, Marcelo de Morais Vitoriano, Francisco de Assis Silva Lima

**Affiliations:** ^1^Department of Endodontics, Course of Dentistry, University of Fortaleza, Fortaleza, CE, Brazil; ^2^Department of Bucco-Maxillofacial Surgery, Course of Dentistry, University of Fortaleza, Fortaleza, CE, Brazil

## Abstract

This case report described the endodontic treatment and decompression of an extensive lesion in the anterior region of the mandible, detected during clinical and radiographic examination, in a patient with a complaint of slight tenderness to palpation in the area of mandibular right lateral incisor and canine. These teeth had been accessed without proper clinical evaluation, and their pulp tissues were exposed. The periodontal tissues were healthy, with no signs of inflammation or fistula. On radiographic examination, a radiolucent lesion with well-defined borders was seen extending from the distal root of mandibular left second premolar to the mesial root of mandibular right second premolar. Central and lateral mandibular left incisors were unresponsive to thermal pulp testing and exhibited coronal discoloration, consistent with a diagnosis of pulp necrosis. Due to persistent discharge from the root canal system during endodontic procedures despite application of intracanal medicament (calcium hydroxide paste), the decision was made to biopsy and decompress the lesion and conclude endodontic treatment. Histopathologic examination revealed a periapical granuloma. After endodontic treatment of the involved teeth, at 4-year clinical and radiographic follow-up, the affected region was almost completely repaired.

## 1. Introduction

Endodontic diagnosis is based on evidence obtained through a thorough history, clinical examination, and radiographic analysis. The interpretation of this evidence provides the dental practitioner with the information needed to establish the correct diagnosis and devise a treatment plan [1, 2].

The development of periapical endodontic lesions is directly associated with migration of microorganisms and/or their byproducts through the root canal system and into the periapical region [3], where they induce an inflammatory response in the periodontal supporting tissues [4, 5].

Periapical granuloma is the most prevalent of the apical lesions. One of the first authors to analyze these lesions was Bhaskar [6], who found that 48% of a series of 2,308 periapical lesions were granulomas. Cysts accounted for 42% of lesions, while other periradicular conditions corresponded to 10% of the analyzed specimens. Nair et al. [7] evaluated 256 lesions and found that 50% were granulomas, 35% were abscesses, and 15% were cysts. More recently, these statistics were confirmed by Çalışkan et al. [8], who analyzed 93 periapical lesions associated with persistent pathosis. In this sample, 72% of lesions were granulomas and 21.5% were radicular cysts. The authors found a positive correlation between lesion size and cystic prevalence, but there were no radiographic signs that could guide differential diagnosis between cystic and granulomatous lesions.

Table 1 provides a brief summary of statistics presented in prior studies of the incidence of granulomas and cysts associated with endodontic treatment.

According to Sugaya and Silva [9], factors such as the shape, location, and speed of expansion of bone lesions, as well as the appearance of the overlying mucosa, are useful signs to guide the differential diagnosis or establish a working diagnosis. Regular expansion with no mucosal discoloration suggests a benign lesion and allows treatment planning with an excellent prognosis.

Okada et al. [10] proposed a noninvasive method to distinguish cysts from granulomas. The authors found that application of computer-aided diagnosis to cone-beam computed tomography images provided results as accurate as histopathological examination. The use of such imaging methods, first reported in 2006 by Simon et al. [11], demonstrated effectiveness in over 94% of cases.

It is well known that granulomas regress with endodontic treatment, thus allowing complete bone repair [12, 13]. Conversely, cystic lesions are exudative and preclude completion of endodontic treatment; conservative therapy must be supplemented by surgical procedures, such as enucleation or decompression.

This article seeks to describe the endodontic treatment and decompression of an extensive lesion in the anterior region of the mandible, detected during clinical and radiographic examination, in a patient with a complaint of slight tenderness to palpation in the area of mandibular right lateral incisor (#42) and canine (#43) in a 23-year-old patient. We also report an error in endodontic diagnosis during the initial management approach.

## 2. Case Report

In March 2010, a 23-year-old woman presented to a primary health care unit in her home town (Ipueiras, state of Ceará, Brazil) with a complaint of tenderness to palpation in the region of teeth #42 and #43. After detection of an extensive periapical lesion in the affected region, both teeth were accessed. However, upon encountering healthy pulp tissue, the local dentist referred the patient for endodontic evaluation at the Universidade de Fortaleza (UNIFOR) School of Dentistry. The past medical history was noncontributory.

**Figure 1:**
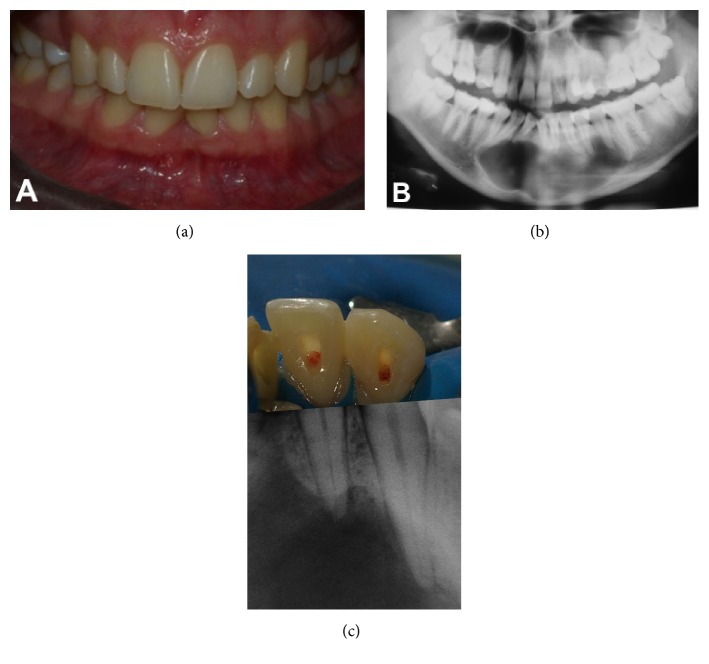
(a) Clinical photograph showing normal periodontal tissues; (b) initial panoramic radiograph; (c) teeth #42 and #43 with open access cavities and exposed pulp prior to instrumentation, demonstrating an error in endodontic diagnosis; relationship between clinical crowns and periapical radiographs.

Initially, we observed slight expansion of the cortical bone in the apical region of #42 and #43, but the periodontal tissues were normal and there was no sinus tract (Figure 1(a)). These teeth had been accessed, and the pulp tissues were clinically visible. Periapical radiographs of the area showed a radiolucent lesion with boundaries extending beyond the dimensions of the periapical film (Figures 1(b) and 1(c)).

Central and lateral left mandibular incisors exhibited coronal discoloration. Teeth #31 to #34 were unresponsive to thermal pulp testing. Percussion testing was negative in all analyzed teeth. There was no tooth mobility. According to the patient, she had sustained avulsion of central mandibular right incisor and a crown fracture in central mandibular left incisor during a motorcycle accident 11 years before.

Extraoral radiographic examination (by panoramic radiography) revealed a large radiolucent lesion, with well-defined borders, extending from the distal root of mandibular #35 to the mesial root of #45 and involving the periapical tissues of all teeth in between.

In light of the radiographic and clinical findings (including coronal discoloration, a clinical sign of pulp necrosis), endodontic treatment of #31 and #32 was indicated. Although #33 and #34 were unresponsive to thermal pulp testing, these teeth did not exhibit other clinical signs of necrosis; thus, endodontic access was deferred. Teeth #42 and #43 had already been accessed and were thus scheduled for endodontic treatment.

Before the start of the procedures, bilateral mental nerve blocks were induced with 2% mepivacaine (Mepiadre 100, DFL Indústria e Comércio SA, Rio de Janeiro, RJ, Brazil). Coronal access was established in the four teeth selected for endodontic treatment and the root canal systems were located. All subsequent steps were performed under rubber dam isolation. The root canal entrances were irrigated copiously with 2.5% sodium hypochlorite (Biodinâmica Química e Farmacêutica Ltda, Ibiporã, Paraná, Brazil), the canals were prepared using crown-down technique, and apical debridement was performed. A Root ZX II apex locator (J Morita) was used to determine working length. After apical debridement, a connection was formed between the root canal system and the apical lesion, flooding the canals with serous discharge.

Despite aspiration, discharge continued to flood the involved teeth. The decision was made to clean and shape the root canals, place intracanal medicaments (calcium hydroxide paste), and prescribe a course of oral amoxicillin (500 mg every 8 hours for 7 days). The teeth were restored provisionally with Cimpat (Septodont, Saint-Maur-des-Fossés, France) and a 7-day follow-up appointment was scheduled. Vigorous aspiration and reapplication of intracanal medicaments were attempted on three more visits, unsuccessfully.

At each visit, a #20 Flex file was used for recapitulation and an F2 file (Protaper) to assist in removal of the intracanal medicament. The root canals were flushed copiously with 2.5% sodium hypochlorite, and final cleaning of the dentin walls was performed with 17% EDTA; both procedures were enhanced by passive ultrasonic irrigation (PUI). At the end of this process, the root canal system was thoroughly suctioned and several attempts were made to dry it with paper points. However, the canals were continually flooded by secretion from the apical lesion, which prevented their obturation. Therefore, calcium hydroxide paste was applied as an intracanal medicament. This procedure was repeated over three visits at 2-week intervals, but was unsuccessful.

**Figure 2:**
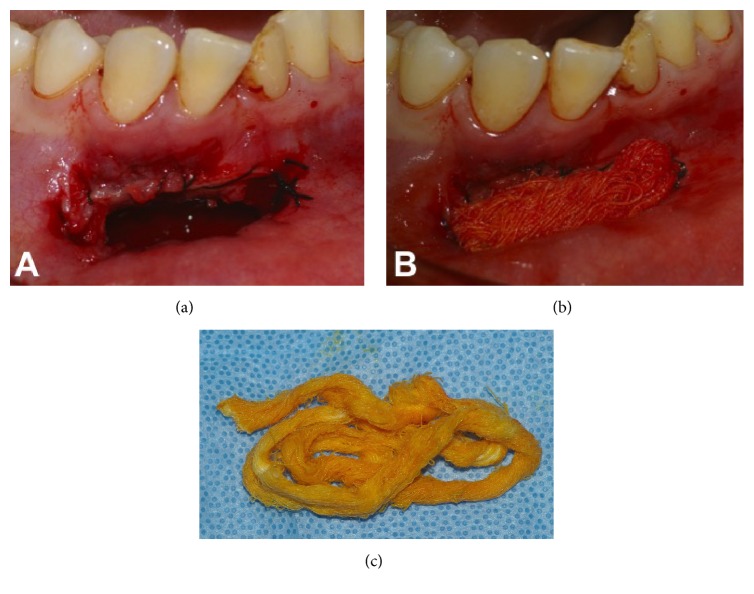
Surgical access through the anterior mandible for decompression of periapical lesion. (a) Cavity after packing (b) with gauze impregnated with antibiotic ointment (c).

On visit 5, as the exudative discharge persisted, the decision was made to decompress the periapical lesion and conclude endodontic treatment. Surgical access to the anterior mandible was obtained with a scalpel and #6 carbide bur mounted on a high-speed handpiece (Figure 2). The procedure was performed in accordance with the principles of the Department of Oral and Maxillofacial Surgery, University of Fortaleza School of Dentistry, under strict biosafety precautions. To prevent soft-tissue migration and sealing of the opening, a running suture was placed on the edges of the lesion. The cavity was irrigated copiously with saline solution, aspirated, and packed with gauze impregnated with a combination corticosteroid/antibiotic ointment (Rifamycin SV/prednisolone acetate, Rifocort®, Medley S.A. Ind Farmacêutica, Campinas, SP, Brazil), in an attempt to prevent opportunistic infections during the early stages of healing and minimize local inflammation. Packing was replaced at 24 and 48 hours to keep the exposed tissues protected. Thereafter, the lesion was simply irrigated with saline solution. The patient was instructed to irrigate the lesion daily and use antiseptic mouthwash to aid infection control. The suture was removed after 7 days.

**Figure 3:**
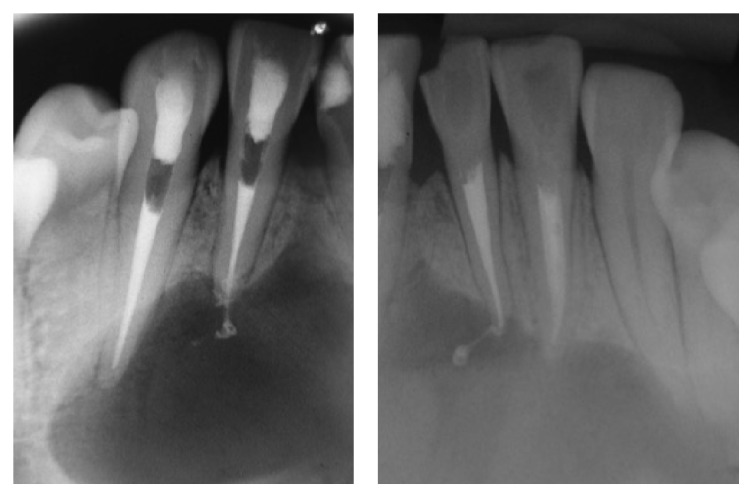
Periapical radiographs obtained immediately after obturation of root canals.

For decompression, bone was exposed and the desired window contour was outlined with a #4 carbide bur. The bone window was carefully removed with Ochsenbein chisels after partial reflection of the overlying soft-tissue lesion. This tissue, approximately 2 mm in diameter and appearing epithelial in origin, was resected and sent to the University of Fortaleza Pathology lab for histopathological examination, which established a diagnosis of granuloma. The root canals were filled during the same visit, immediately after decompression. Briefly, the canals were cleaned and shaped to 1 mm short of full length, using Protaper® rotary instrumentation (Dentsply-Maillefer, Ballaigues, Switzerland), followed by obturation with Protaper F2 gutta-percha cones (Dentsply-Maillefer) and Grossman's cement (Endofill, Dentsply Indústria e Comércio Ltda, Petrópolis, RJ, Brazil), using the lateral condensation technique (Figure 3). The access cavities were then sealed with composite resin (Filtek Z250, 3M ESPE Dental Products, St Paul, MN, USA).

**Figure 4:**
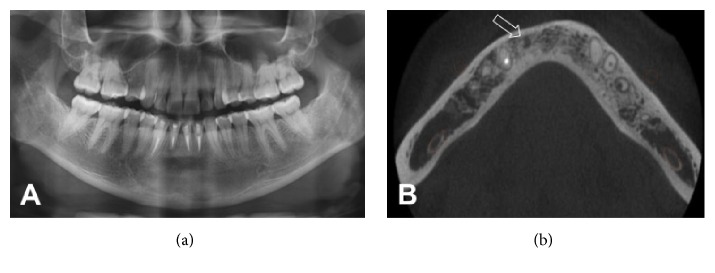
(a) Panoramic radiograph obtained at 4-year follow-up. Bone repair is clearly visible; (b) cone-beam computed tomography image (axial slice), demonstrating bone repair. A possible fibrotic bone scar is visible (arrow).

Initially, the patient presented for monthly follow-up visits. At the first visit, #33 and #34 were found to respond positively to thermal pulp testing. Three months after the procedure, the patient moved to a distant location and was lost to follow-up for 4 years. When she resumed contact, plain radiographs (Figure 4(a)) were obtained and computed tomography was performed (Figure 4(b)). These imaging studies revealed near-complete repair of the bone lesion, and the patient was completely free of signs and symptoms of inflammation.

## 3. Discussion

Several studies on errors in endodontic diagnosis have been published in the literature [1, 2], and much research has been conducted on tools and methods to assist in their detection [12, 17]. Errors in endodontic diagnosis may be associated with the radiographic appearance of lesions that suggest apical involvement but are not of endodontic origin [18], or may be attributable to human error on the part of the professional [18]. A lack of attention to clinical and radiographic manifestations can prevent proper case planning.

As pointed out by Sirotheau Corrêa Pontes et al. [2], lesions not of endodontic origin but located near the root apex may compromise blood circulation through the neurovascular bundle that supplies the pulp tissues, thus leading to pulp necrosis [19]. On the basis of this principle, endodontists should take into account clinical aspects, radiographic findings, and the patient's dental history [20] and attempt to ascertain whether absence of response to thermal pulp testing is a transient phenomenon or attributable to another etiology, such as transient paresthesia secondary to nerve compression [21].

In the case reported herein, teeth #33 and #34 were initially unresponsive to thermal pulp testing, but as there was no coronal discoloration, the chosen course of treatment consisted of watchful waiting and later reassessment of pulp sensitivity [20]. At 30-day clinical follow-up, these teeth were found to be responsive to thermal pulp testing, thus demonstrating their viability and that the initial response was unreliable [21].

Granulomas are usually asymptomatic and develop as the result of pulp necrosis. Granulomatous tissue is essentially composed of a chronic inflammatory infiltrate in association with reparative elements, which takes the place of resorbed bone [22]. The extent of these lesions is variable. Growth of periapical lesions has been attributed to factors such as increased hydrostatic pressure within the confined fluid, which promotes increased osteoclast activity [12]. According to Saraf et al. [5], extensive granulomas have the potential for cystic transformation, which would make the case an endodontic treatment failure. In the case presented herein, the lesion likely developed secondary to the motorcycle accident in which the patient had been involved many years before. Most probably, the pulp tissue of teeth #31 and #32 was not unable to withstand the trauma and necrosis and then set in, which allowed a smoldering infection to develop and subsequently lead to a chronic, asymptomatic lesion.

Decompression is indicated in large cystic lesions with involvement of anatomic structures such as the maxillary sinus, nasal cavity, or skeletal architecture [23]. Our patient had near-total involvement of the anterior mandible. The purposes of decompression are to debulk the lesion, thus enabling conservative tissue resection [23], and to allow completion of endodontic treatment in cases with pulp and periapical tissue involvement [12], as occurred in our patient. A recent report in the literature described use of the EndoVac system as an alternative for decompression of a large periapical lesion of endodontic origin [12]. This lesion was probably a granuloma, as repair was achieved without the need for additional surgical intervention.

It bears stressing that proper endodontic diagnosis is based on a detailed analysis of clinical aspects (pulp vitality testing) and radiographic examination of periapical tissues and should take into account the patient's past medical history as well as the natural history of the current complaint [1, 5]. The core purpose of endodontic treatment is to address restorative issues present in the affected tooth, thus enabling recovery of cosmetics and function, eliminating any signs and symptoms of inflammation and allowing bone repair of apical lesions [10, 14]. Therefore, it is clear that the endodontist must ascertain which type of lesion is affecting the bone, which is only possible through biopsy and histopathological examination.

Although some studies have reported the nonsurgical treatment of cystic lesions [24, 25], the success of such conservative approaches requires certain clinical conditions, including a dry environment, free of persistent discharge. In the case described herein, nonsurgical treatment was a priority, but persistent exudation prevented completion of therapy. This prompted us to plan a supplemental surgical phase of treatment. Given the extent of the lesion, we decided to perform decompression, immediate obturation of the root canal system and clinical and radiographic follow-up. Enucleation of the lesion followed by apicectomy could further weaken the bone structure, under the risk of mandibular fracture. Corroborating our approach, the aforementioned authors state that treatment success is directly associated with elimination of root canal infection [24] and with the duration of follow-up [25].

However, excisional biopsy cannot and should not be performed in every tooth affected by extensive periapical lesions, not least because, in most cases, these lesions resolve after appropriate endodontic treatment [15, 26]. Thus, clinical and radiographic follow-up are clearly necessary to determine whether clinical signs of inflammation (swelling, pain, and fistula) are absent or resolving and whether bone repair is taking place [26, 27]. In lesions that exhibit persistent exudation between visits, preventing conclusion of endodontic treatment, supplemental approaches (including surgical procedures) are needed to eliminate the offending factor and allow tissue repair to proceed.

## Figures and Tables

**Table 1:** Distribution of chronic periapical lesions as reported in various studies.

Study	Granuloma (%)	Cyst (%)	Abscess (%)	Other (%)
Bhaskar, 1966 [6]	48.0	42.0	—	10.0
Nair et al., 1996 [7]	50.0	15.0	35.0	—
Ricucci et al., 2006 [14]	61.4	17.5	21.1	—
Becconsall-Ryan et al., 2010 [15]	59.7	29.2	—	—
Love and Firth, 2009 [4]	77.0	18.0	3.0	2.0
Koivisto et al., 2012 [16]	40.4	33.1		26.5
Saraf et al., 2014 [5]	66.7	10.0	6.7	—
Çalışkan et al., 2016 [8]	72.0	21.5	4.3	2.2
